# Supplementary material for: Improved designs for pET expression plasmids increase protein production yield in Escherichia coli
Source: Commun Biol. 2020 May 7;3:214. doi: 10.1038/s42003-020-0939-8 (PMC7205610; doi:10.1038/s42003-020-0939-8)
Supplement: Supplementary file 1 — Supplementary information [file 42003_2020_939_MOESM1_ESM.pdf]

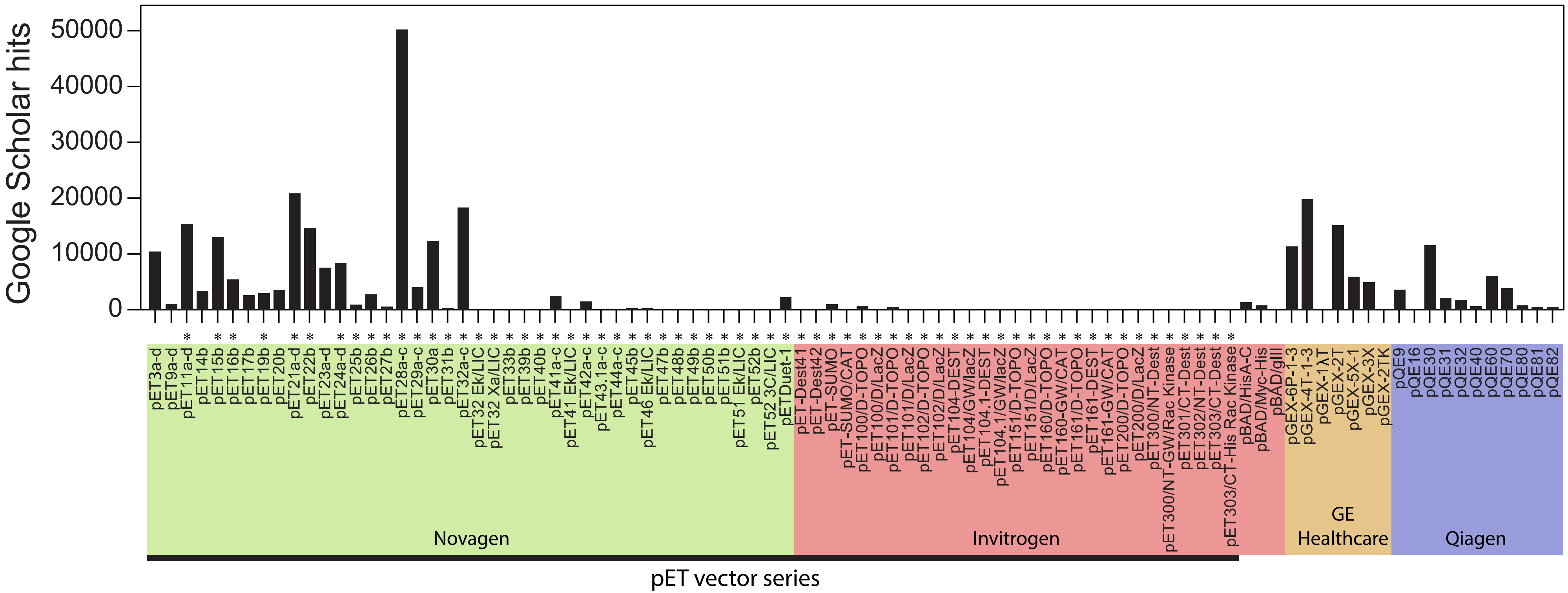

Supplementary Figure 1. Popular recombinant protein expression plasmids and their recorded use in publication.

The pET plasmid series is the most popular when compared to alternatives, such as the pGEX, pQE or pBAD plasmids. pET vectors have a total of 220,000 entries in the publication record (Google Scholar). Of the pET plasmids, pET28a-c is the most widely used, recording over 50,000 recorded entries, of which pET28a alone accounts for 40,000 entries. An asterix (*) next to a plasmid name denotes those for which the φ10 (T7)-promoter is a truncated variant of the consensus φ10 (T7)-promoter

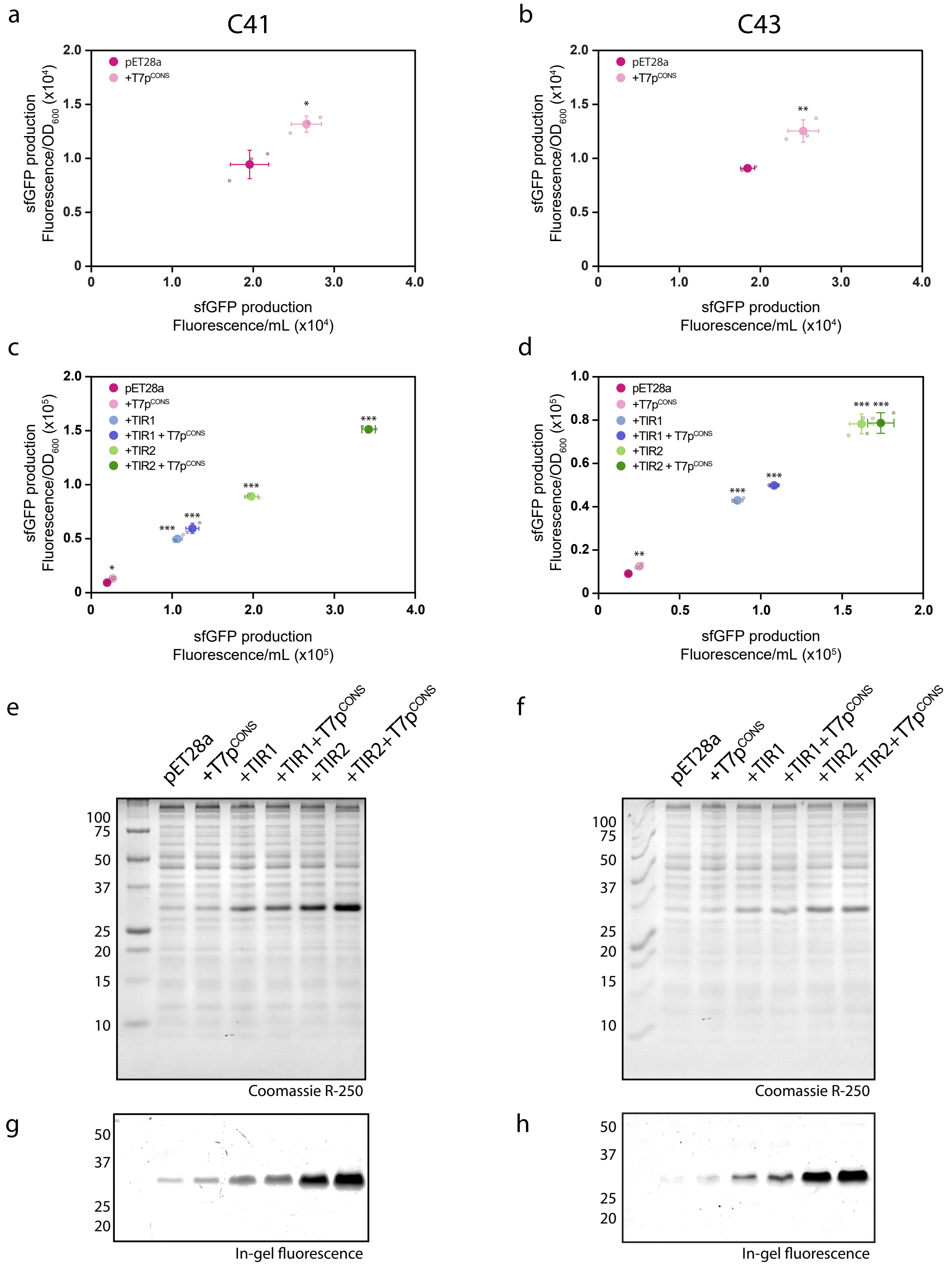

Supplementary Figure 2. Addition of T7p^{CONS} and TIR-1 and TIR-2 increase production yields of His$_6$-TPS-sfGFP in the Walker strains, C41 and C43.

Top panels reveal that the addition of the T7p^{CONS} to pET28a His$_6$-TPS-sfGFP, increases efficiency by 1.44- and 1.42-fold for **a.** C41 and **b.** C43, respectively. Further enhancement was observed when introducing TIR-1 and TIR-2 **c.** and **d.** For the combination of T7p^{CONS} and TIR-1 and TIR-2, the C41 strain showed increased efficiency. Data presented as mean ± s.d. (n = 3). An asterix denotes a statistically significant difference of * $p < 0.05$, ** $p < 0.01$ and *** $p < 0.001$ relative to pET28a (two-tailed Student's t-test). **e.** and **f.** SDS-PAGE is represented from one replicate with normalised loading of 0.05 OD_{600} units. **g andh.** In-gel fluorescence. Gels are identical to e and f respectively. Images taken prior to coomassie staining.

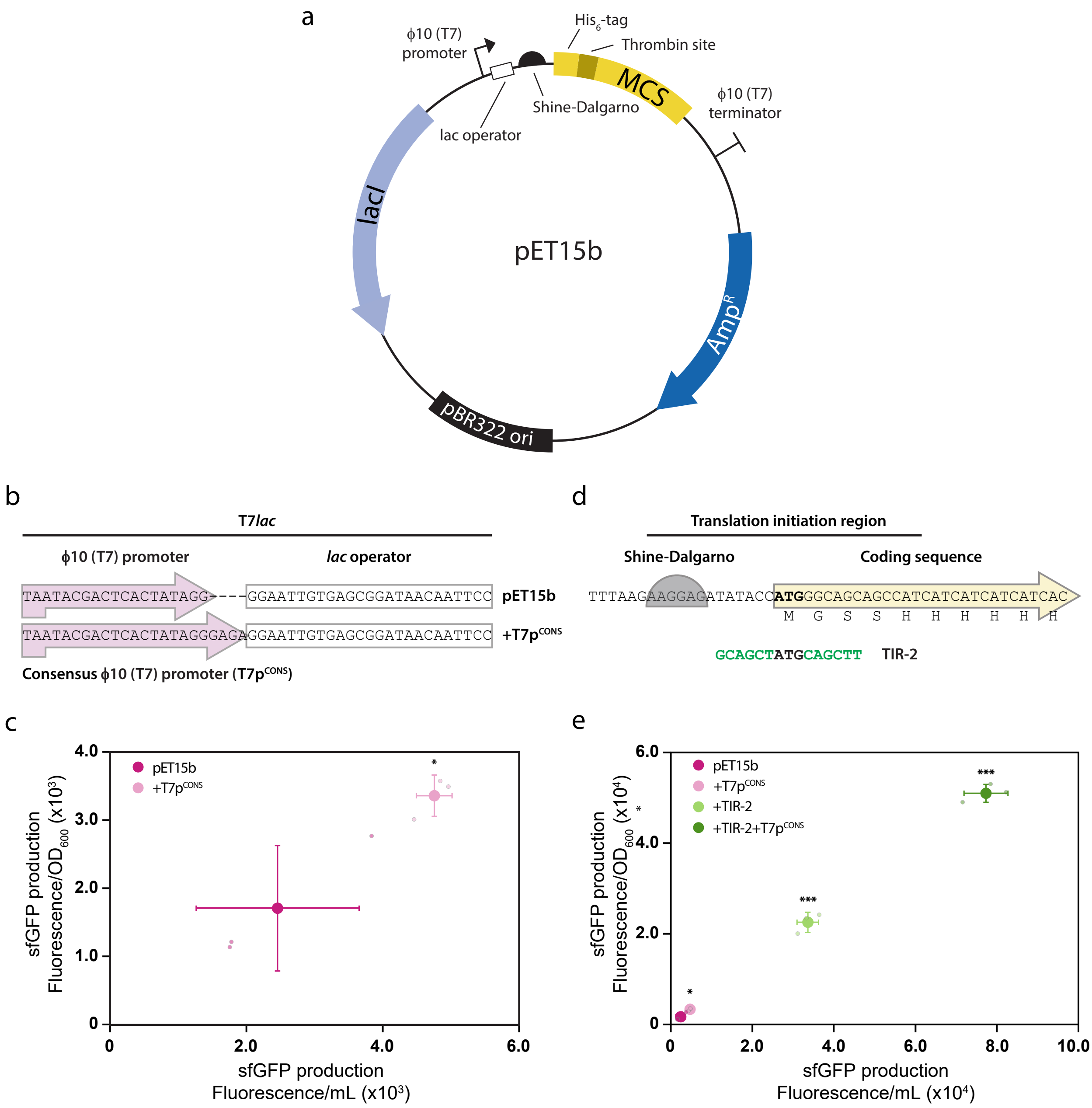

Supplementary Figure 3. Salient features of pET15b, design flaws and optimisation.

a. Genetic elements present in pET15b include the ϕ10 (T7)-promoter and the lac operator, as well as the TIR encompassing the SD and coding sequence. **b.** The ϕ10 (T7)-promoter in pET15b is the same as pET28a; a truncated variant of the consensus ϕ10 (T7)-promoter (T7p^{CONS}). **c.** Inclusion of the T7p^{CONS} results in a three-fold increase in His$_6$-TPS-sfGFP levels. **d.** Addition of TIR-2 or the combination of TIR-2 and T7p^{CONS} resulted in up to **e.** a 28 and 65-fold increase in His$_6$-TPS-sfGFP production levels, respectively. Data presented as mean ± s.d. ($n = 3$). An asterix denotes a statistically significant difference of * $p < 0.05$, * $p < 0.05$ and *** $p < 0.001$ relative to pET28a (two-tailed Student's t-test).

a

b

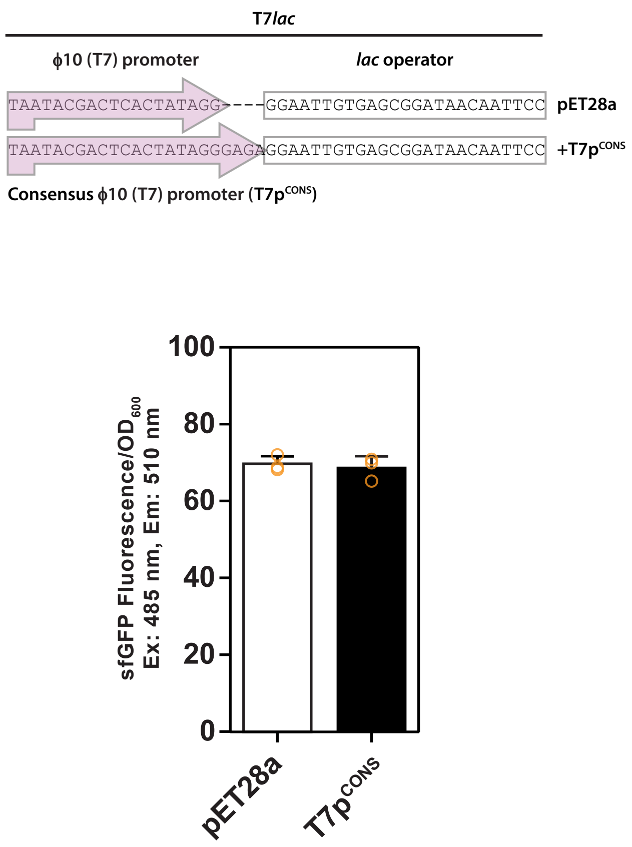

Supplementary Figure 4. Normalised sfGFP expression prior to IPTG induction.

a. Addition of a T7p^{CONS} in pET28a does not influence the adjacent lac operator sequence and **b.** does not promote an increase in the levels of His$_6$-TPS-sfGFP prior to IPTG induction. Data presented as mean ± s.d. (n = 3).

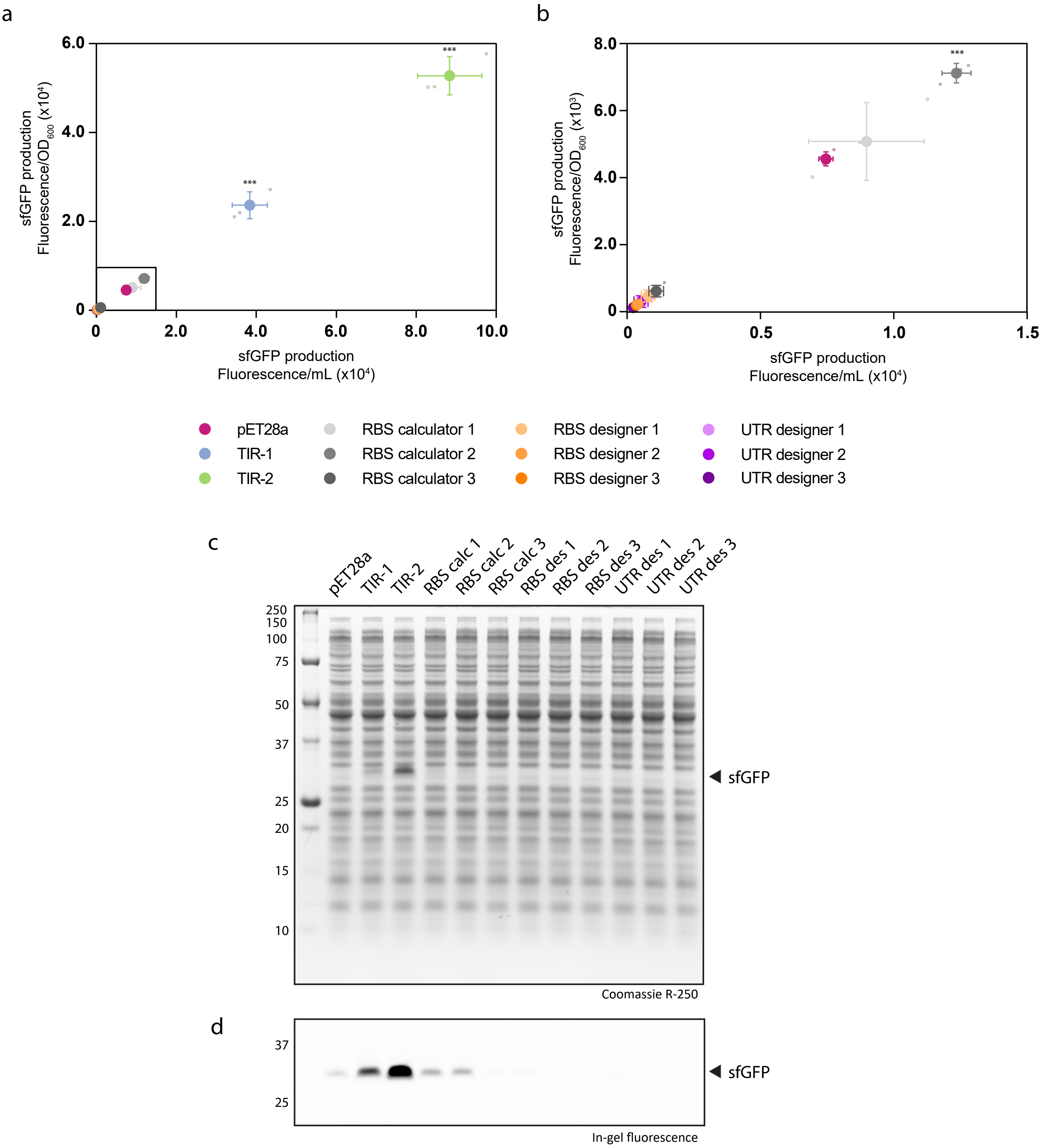

Supplementary Figure 5. Benchmarking synthetically evolved TIRs to *in silico* predicted TIRs.

Previously identified TIR-1 and TIR-2 clones were engineered into the pET28a-His_6-TPS-sfGFP plasmid. In addition, nine *in silico* predicted TIRs were also introduced into the pET28a-His_6-TPS-sfGFP plasmid. *In silico* predictions were created using RBS calculator, RBS designer and UTR designer. **a.** Fluorescence assays were carried out for TIR-1, TIR-2 and nine *in silico* predicted TIRs. Both TIR-1 and TIR-2 variants displayed greater expression levels than the standard pET28a $TIR^{UNEVOLVED}$. **b.** Boxed region from a. Only two of nine *in silico* predicted TIRs showed expression levels higher than the standard pET28a $TIR^{UNEVOLVED}$. All other calculated TIRs exhibited negligible fluorescence. Data presented as mean ± s.d. (n = 3). An asterix denotes a statistically significant difference of *** $p < 0.001$ relative to pET28a $TIR^{UNEVOLVED}$ (two-tailed Student's t-test). **c.** SDS-PAGE of one replicate with normalised loading of 0.05 OD_{600} units. **d.** In-gel fluorescence. Gel is identical to c. Image taken prior to coomassie staining.

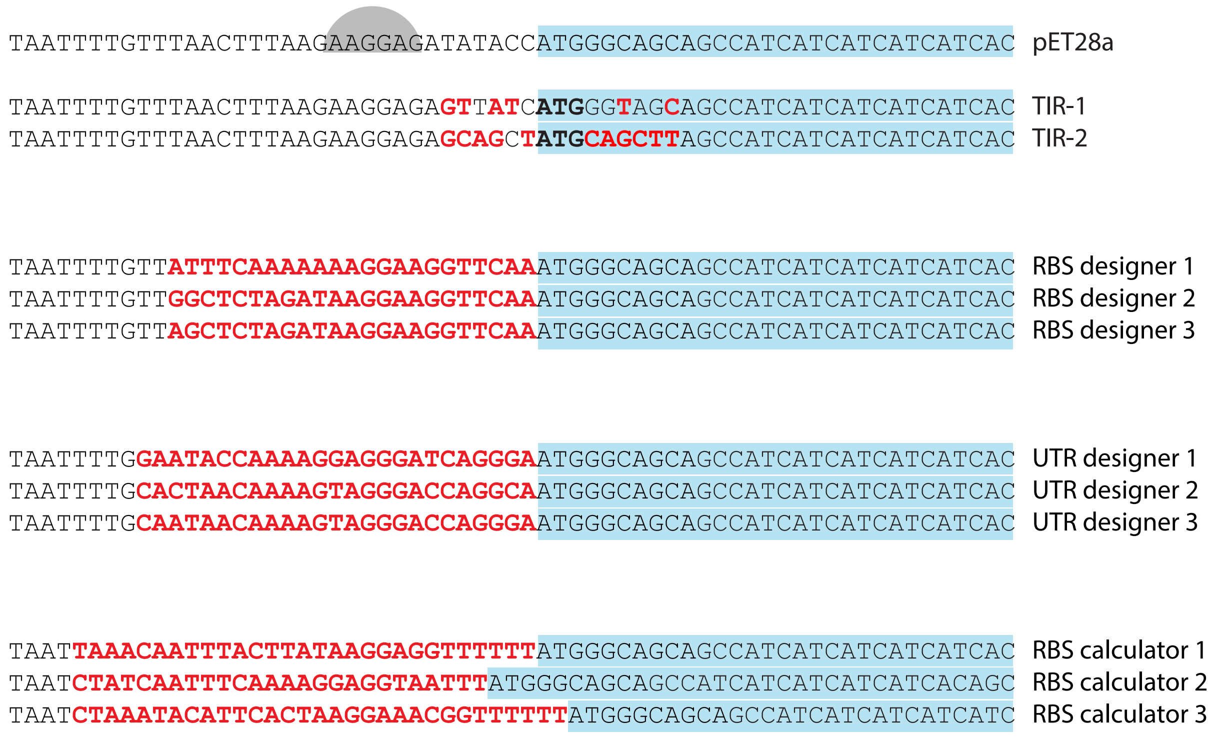

Supplementary Figure 6. Experimentally determined TIRs and *in silico* calculated TIRs.

The TIR region of pET28a is depicted. The SD is shown as a grey semi-circle and the coding sequence is boxed in blue. Below are the corresponding sequences obtained for the experimentally determined TIR-1 and TIR-2; sequence highlighted in red indicates the nucleotides that were mutated during the synthetic evolution process. Three *in silico* predicted TIRs were obtained from three calculators. For clarity, the identified sequences and the position of insertion in pET28a, are shown in red.

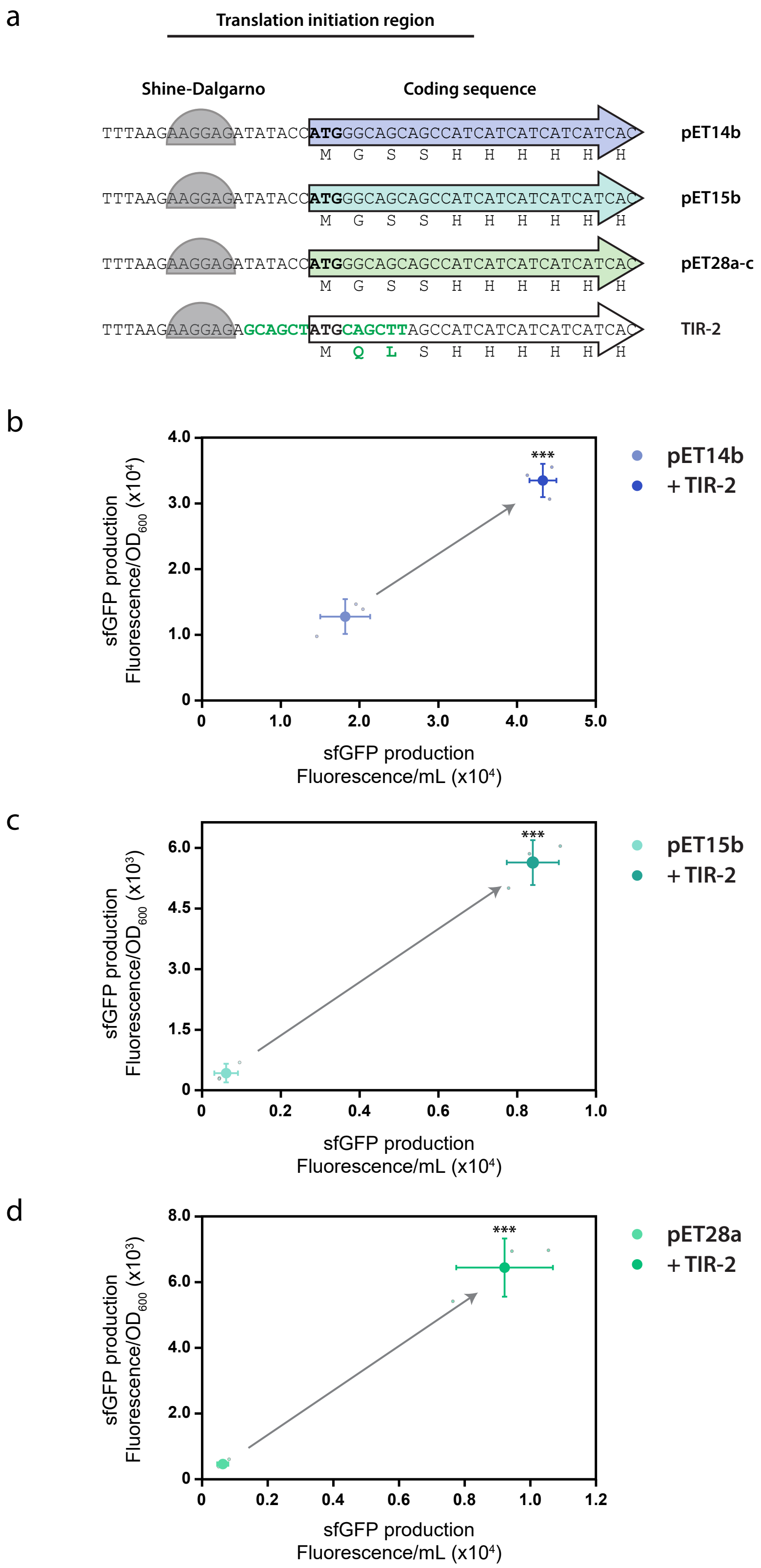

Supplementary Figure 7. Complementation of enhanced expression in alternative pET plasmids.

a. The TIR regions of the pET14b, pET15b and pET28a are 100% identical, as they possess the same SD, spacer, His_6-tag and thrombin protease site. sfGFP was cloned into pET14b and pET15b using the identical NdeI restriction site immediately following the thrombin protease site as found in pET28a; a feature shared between all three plasmids. sfGFP expression was significantly enhanced when TIR-2 was introduced into **b.** pET14b **c.** pET15b and **d.** pET28a. Data presented as mean ± s.d. (n = 3). An asterix denotes a statistically significant difference of *** $p < 0.001$ (two-tailed Student's t-test).

```
GTAGAGGATCGAGATCTCGATCCCGCGAAATTAATACGACTCACTATAGGGGAATTGTGAGCGGATAACAATTCCCCT
CTAGAAATAATTTTGTTTAACTTTAAGAAGGAGATATACCATGGGCAGCAGCCATCATCATCATCATCACAGCAGCGG
CCTGGTGCCGCGCGGCAGCCATATGAGCAAAGGAGAAGAACTTTTCACTGGAGTTGTCCCAATTCTTGTTGAATTAGA
TGGTGATGTTAATGGGCACAAATTTTCTGTCCGTGGAGAGGGTGAAGGTGATGCTACAAACGGAAAACTCACCCTTAA
ATTTATTTGCACTACTGGAAAACTACCTGTTCCGTGGCCAACACTTGTCACTACTCTGACCTATGGTGTTCAATGCTT
TTCCCGTTATCCGGATCACATGAAACGGCATGACTTTTTCAAGAGTGCCATGCCCGAAGGTTATGTACAGGAACGCAC
TATATCTTTCAAAGATGACGGGACCTACAAGACGCGTGCTGAAGTCAAGTTTGAAGGTGATACCCTTGTTAATCGTAT
CGAGTTAAAAGGTATTGATTTTAAAGAAGATGGAAACATTCTCGGACACAAACTGGAGTACAACTTTAACTCACACAA
TGTATACATCACGGCAGACAAACAAAAGAATGGAATCAAAGCTAACTTCAAAATTCGCCACAACGTTGAAGATGGTTC
CGTTCAACTAGCAGACCATTATCAACAAAATACTCCAATTGGCGATGGCCCTGTCCTTTTACCAGACAACCATTACCT
GTCGACACAATCTGTTCTTTCGAAAGATCCCAACGAAAAGCGTGACCACATGGTCCTTCTTGAGTTTGTAACTGCTGC
TGGGATTACACATGGCATGGATGAACTCTACAAATAACTCGAGCACCACCACCACCACCACTGAGATCCGGCTGCTAA
CAAAGCCCGAAAGGAAGCTGAGTTGGCTGCTGCCACCGCTGAGCAATAACTAGCATAACCCCTTGGGGCCTCTAAACG
GGTCTTGAGGGGTTTTTTGCTGAAAGGAGGAACTATATCCGGATTGGCGA
```

Supplementary Figure 8. Sequence of pET28a His_6-TPS-sfGFP

The ϕ10 (T7)-promoter is depicted by the pink arrow. Directly adjacent is the lac operon (boxed). The Shine-Dalgarno (SD) sequence is shown as a semi-circle followed by a linker of seven nucleotides. The coding sequence (blue box) encodes an N-terminal His_6-tag and thrombin protease site followed by sfGFP (green box). sfGFP was cloned into pET28a using the NdeI and XhoI restriction sites. The ϕ10 (T7)-terminator is boxed in grey.

```
GTAGAGGATCGAGATCTCGATCCCGCGAAATTAATACGACTCACTATAGGGGAATTGTGAGCGGATAACAATTCCCCT
CTAGAAATAATTTTGTTTAACTTTAAGAAGGAGATATACCATGGGCAGCAGCCATCATCATCATCATCACAGCAGCGG
CCTGGTGCCGCGCGGCAGCCATATGAGCAAAGGAGAAGAACTTTTCACTGGAGTTGTCCCAATTCTTGTTGAATTAGA
TGGTGATGTTAATGGGCACAAATTTTCTGTCCGTGGAGAGGGTGAAGGTGATGCTACAAACGGAAAACTCACCCTTAA
ATTTATTTGCACTACTGGAAAACTACCTGTTCCGTGGCCAACACTTGTCACTACTCTGACCTATGGTGTTCAATGCTT
TTCCCGTTATCCGGATCACATGAAACGGCATGACTTTTTCAAGAGTGCCATGCCCGAAGGTTATGTACAGGAACGCAC
TATATCTTTCAAAGATGACGGGACCTACAAGACGCGTGCTGAAGTCAAGTTTGAAGGTGATACCCTTGTTAATCGTAT
CGAGTTAAAAGGTATTGATTTTAAAGAAGATGGAAACATTCTCGGACACAAACTGGAGTACAACTTTAACTCACACAA
TGTATACATCACGGCAGACAAACAAAAGAATGGAATCAAAGCTAACTTCAAAATTCGCCACAACGTTGAAGATGGTTC
CGTTCAACTAGCAGACCATTATCAACAAAATACTCCAATTGGCGATGGCCCTGTCCTTTTACCAGACAACCATTACCT
GTCGACACAATCTGTTCTTTCGAAAGATCCCAACGAAAAGCGTGACCACATGGTCCTTCTTGAGTTTGTAACTGCTGC
TGGGATTACACATGGCATGGATGAACTCTACAAATGAAATAGGAGGTCCTCCTATTTCAATTCAACATTTCCGTGTCG
CCCTTATTCCCTTTTTTGCGGCATTTTGCCTTCCTGTTTTTGCTCACCCAGAAACGCTGGTGAAAGTAAAAGATGCTG
AAGATCAGTTGGGTGCACGAGTGGGTTACATCGAACTGGATCTCAACAGCGGTAAGATCCTTGAGAGTTTTCGCCCCG
AAGAACGTTTTCCAATGATGAGCACTTTTAAAGTTCTGCTATGTGGCGCGGTATTATCCCGTATTGACGCCGGGCAAG
AGCAACTCGGTCGCCGCATACACTATTCTCAGAATGACTTGGTTGAGTACTCACCAGTCACAGAAAAGCATCTTACGG
ATGGCATGACAGTAAGAGAATTATGCAGTGCTGCCATAACCATGAGTGATAACACTGCGGCCAACTTACTTCTGACAA
CGATCGGAGGACCGAAGGAGCTAACCGCTTTTTTGCACAACATGGGGGATCATGTAACTCGCCTTGATCGTTGGGAAC
CGGAGCTGAATGAAGCCATACCAAACGACGAGCGTGACACCACGATGCCTGTAGCAATGGCAACAACGTTGCGCAAAC
TATTAACTGGCGAACTACTTACTCTAGCTTCCCGGCAACAATTAATAGACTGGATGGAGGCGGATAAAGTTGCAGGAC
CACTTCTGCGCTCGGCCCTTCCGGCTGGCTGGTTTATTGCTGATAAATCTGGAGCCGGTGAGCGTGGGTCTCGCGGTA
TCATTGCAGCACTGGGGCCAGATGGTAAGCCCTCCCGTATCGTAGTTATCTACACGACGGGGAGTCAGGCAACTATGG
ATGAACGAAATAGACAGATCGCTGAGATAGGTGCCTCACTGATTAAGCATTGGTAACTCGAGCACCACCACCACCACC
ACTGAGATCCGGCTGCTAACAAAGCCCGAAAGGAAGCTGAGTTGGCTGCTGCCACCGCTGAGCAATAACTAGCATAAC
CCCTTGGGGCCTCTAAACGGGTCTTGAGGGGTTTTTTGCTGAAAGGAGGAACTATATCCGGAT
```

Supplementary Figure 9. Sequence of the pET28a His$_6$-TPS-sfGFP-hp-Amp^R expression casette

The pET28a-His$_6$-TPS-sfGFP-hp-Amp^R expression cassette is shown. The ϕ10 (T7)-promoter is depicted by the pink arrow. Directly adjacent is the lac operon (boxed). The Shine-Dalgarno sequence is shown as a semi-circle followed by a linker of seven nucleotides. The coding sequence (blue box) encodes an N-terminal His$_6$-tag and thrombin protease site followed by sfGFP (green box). The translational coupling device (hp; weak coupling 1 - pink box) was inserted directly after and in frame with sfGFP. The translational coupling device encodes an RNA hairpin that does not encode a protein but places the adjacent β-lactamase (purple) in close proximity to sfGFP. The ϕ10 (T7)-terminator is boxed in grey.

GTAGAGGATCGAGATCTCGATCCCGCGAAATTAATACGACTCACTATAGGGGAATTGTGAGCGGATAACAATTCCCCT
CTAGAAATAATTTTGTTTAACTTTAAGAAGGAGATATACCATGGGCAGCAGCCATCATCATCATCATCACAGCAGCGG
CCTGGTGCCGCGCGGCAGCCATATGGGTGCAAGCCGTCTGTATACCCTGGTTCTGGTGCTGCAGCCGCAACGTGTTCT
GCTGGGTATGAAAAAACGTGGTTTTGGTGCAGGTCGTTGGAATGGTTTTGGTGGTAAAGTTCAGGAAGGCGAAACCAT
TGAAGATGGTGCACGTCGTGAACTGCAGGAAGAAAGCGGTCTGACCGTTGATGCACTGCATAAAGTTGGCCAGATTGT
GTTTGAATTTGTGGGTGAACCGGAACTGATGGATGTTCATGTGTTTTGCACCGATAGCATTCAGGGTACACCGGTTGA
ATCTGATGAAATGCGTCCGTGTTGGTTTCAGCTGGATCAGATTCCGTTTAAAGATATGTGGCCTGATGATAGCTATTG
GTTTCCGCTGCTGCTGCAGAAAAAGAAATTCCATGGCTACTTCAAATTTCAGGGCCAGGATACCATTCTGGATTATAC
CCTGCGTGAAGTTGATACCGTGTAATAAGCGGCCGCACTCGAGCACCACCACCACCACCACTGAGATCCGGCTGCTAA
CAAAGCCCGAAAGGAAGCTGAGTTGGCTGCTGCCACCGCTGAGCAATAACTAGCATAACCCCTTGGGGCCTCTAAACG
GGTCTTGAGGGGTTTTTTGCTGAAAGGAGGAACTATATCCGGATTGGCGA

Supplementary Figure 10. Sequence of pET28a His$_6$-TPS-MTH1

The ϕ10 (T7)-promoter is depicted by the pink arrow. Directly adjacent is the lac operon (boxed). The Shine-Dalgarno sequence is shown as a semi-circle followed by a linker of seven nucleotides. The coding sequence (blue box) encodes an N-terminal His$_6$-tag and thrombin protease site followed by MTH1 (green box). MTH1 was cloned into pET28a between the NdeI and NotI restriction sites. The ϕ10 (T7)-terminator is boxed in grey.

```
GTAGAGGATCGAGATCTCGATCCCGCGAAATTAATACGACTCACTATAGGGGAATTGTGAGCGGATAACAATTCCCCT
CTAGAAATAATTTTGTTTAACTTTAAGAAGGAGATATACCATGGGCAGCAGCCATCATCATCATCATCACAGCAGCGG
CCTGGTGCCGCGCGGCAGCCATATGGTGGAAGGACCAGGCTGTACTCTGAATGGAGAGAAGATTCGCGCGCGGGTGCT
CCCGGGCCAGGCGGTGACCGGCGTGCGGGGAAGCGCTCTGCGGAGTCTGCAGGGCCGCGCCTTGCGGCTCGCAGCCTC
CACGGTTGTGGTCTCCCCGCAGGCTGCTGCACTGAATAATGATTCCAGCCAGAATGTCTTGAGCCTGTTTAATGGATA
TGTTTACAGTGGCGTGGAAACTTTGGGGAAGGAGCTCTTTATGTACTTTGGACCAAAAGCTTTACGGATTCATTTCGG
AATGAAAGGCTTCATCATGATTAATCCACTTGAGTATAAATATAAAAATGGAGCTTCTCCTGTTTTGGAAGTGCAGCT
CACCAAAGATTTGATTTGTTTCTTTGACTCATCAGTAGAACTCAGAAACTCAATGGAAAGCCAACAGAGAATAAGAAT
GATGAAAGAATTAGATGTATGTTCACCTGAATTTAGTTTCTTGAGAGCAGAAAGTGAAGTTAAAAAACAGAAAGGCCG
GATGCTAGGTGATGTGCTAATGGATCAGAACGTATTGCCTGGAGTAGGGAACATCATCAAAAATGAAGCTCTCTTTGA
CAGTGGTCTCCACCCAGCTGTTAAAGTTTGTCAATTAACAGATGAACAGATCCATCACCTCATGAAAATGATACGTGA
TTTCAGCATTCTCTTTTACAGGTGCCGTAAAGCAGGACTTGCTCTCTCTAAACACTATAAGGTTTACAAGCGTCCTAA
TTGTGGTCAGTGCCACTGCAGAATAACTGTGTGCCGCTTTGGGGACAATAACAGAATGACATATTTCTGTCCTCACTG
TCAATAACTCGAGCACCACCACCACCACCACTGAGATCCGGCTGCTAACAAAGCCCGAAAGGAAGCTGAGTTGGCTGC
TGCCACCGCTGAGCAATAACTAGCATAACCCCTTGGGGCCTCTAAACGGGTCTTGAGGGGTTTTTTGCTGAAAGGAGG
AACTATATCCGGATTGGCGA
```

Supplementary Figure 11. Sequence of pET28a His$_6$-TPS-Neil3

The ϕ10 (T7)-promoter is depicted by the pink arrow. Directly adjacent is the lac operon (boxed). The Shine-Dalgarno sequence is shown as a semi-circle followed by a linker of seven nucleotides. The coding sequence (blue box) encodes an N-terminal His$_6$-tag and thrombin protease site followed by Neil3 (green box). Neil3 was cloned into pET28a between the NdeI and XhoI restriction sites. The ϕ10 (T7)-terminator is boxed in grey.

a

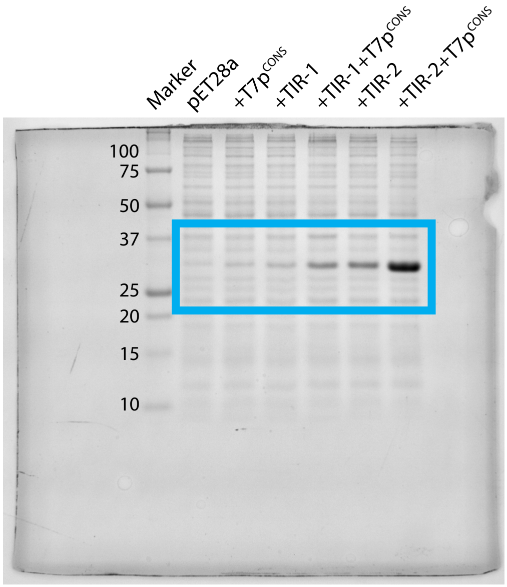

b

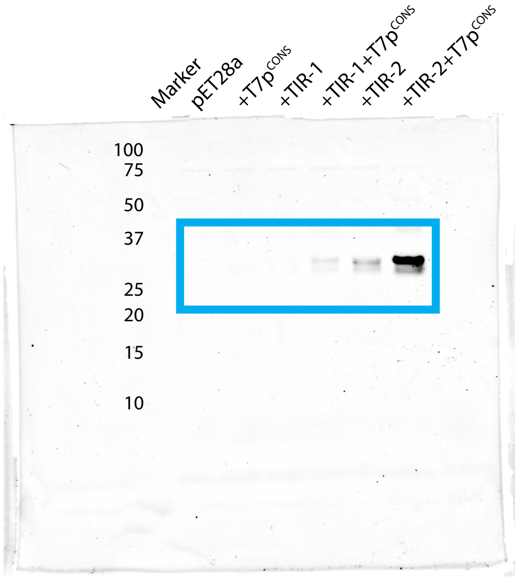

Supplementary Figure 12. Uncropped gels used in Figure 4b

All lanes are labelled according to the figure within the main text. Marker is indicated. Blue boxes indicate cropped portions used in Figure 4b.
a. Coomassie R-250 stained gel
b. In-gel fluorescence (Gel is identical to a. Image taken prior to coomassie staining)

a

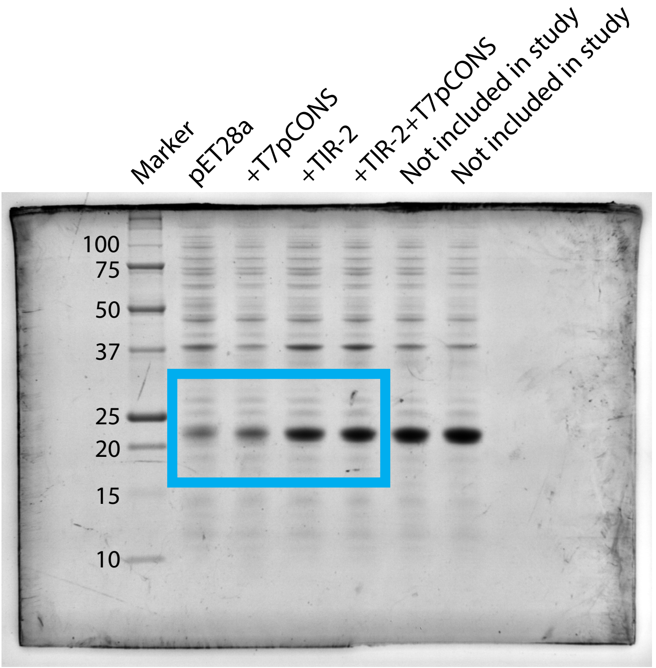

b

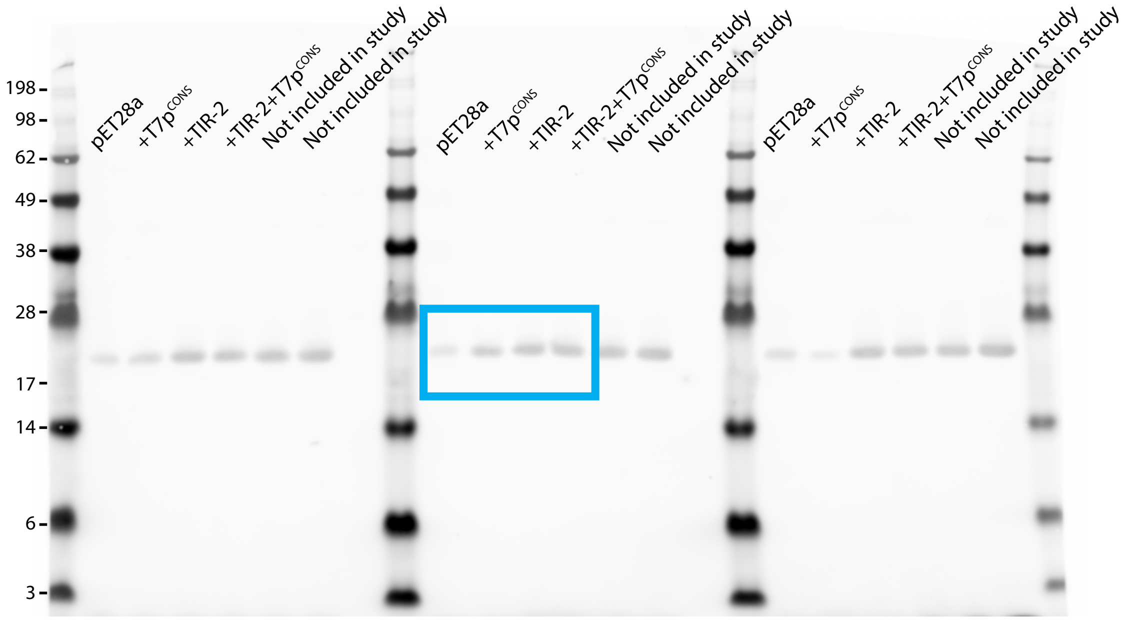

Supplementary Figure 13. Uncropped gel and Western blot used in Figure 4c

All lanes are labelled according to the figure within the main text. Marker is indicated. Blue boxes indicate cropped portions used in Figure 4c.

a. Coomassie R-250 stained gel for one replicate

b. Western blot detected by 1° Anti-MTH1 and 2° IR-700

a

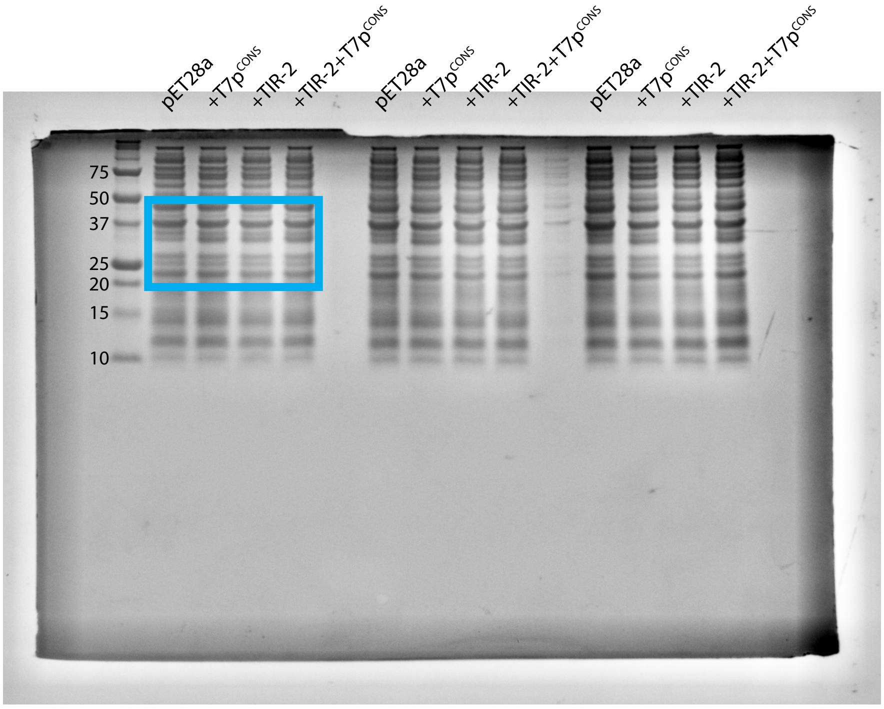

b

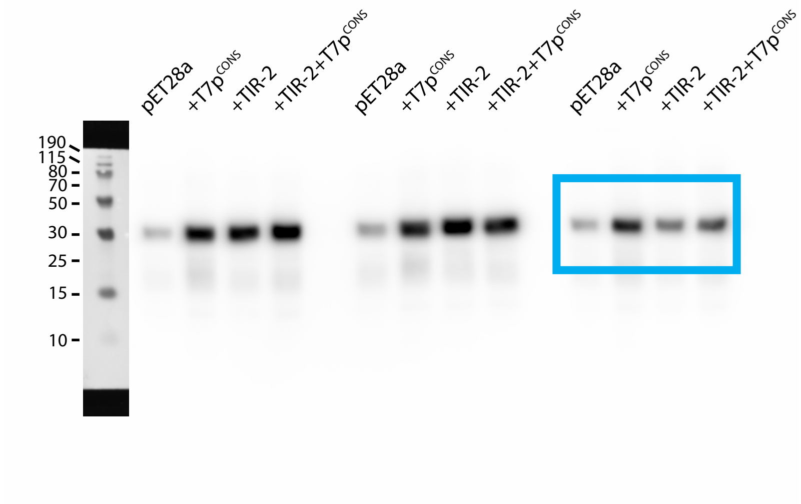

Supplementary Figure 14. Uncropped gel and Western blot used in Figure 4d

All lanes are labelled according to the figure within the main text. Marker is indicated. Blue boxes indicate cropped portions used in Figure 4d

a. Coomassie R-250 stained gel for one replicate

b. Western blot detected by 1° Anti-His-HRP

a

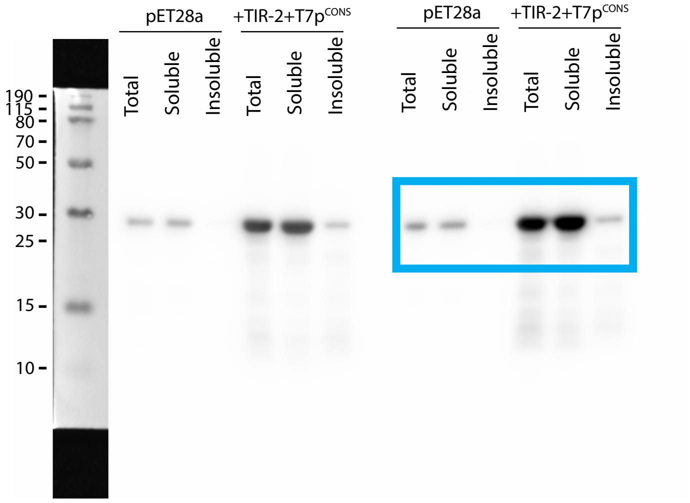

b

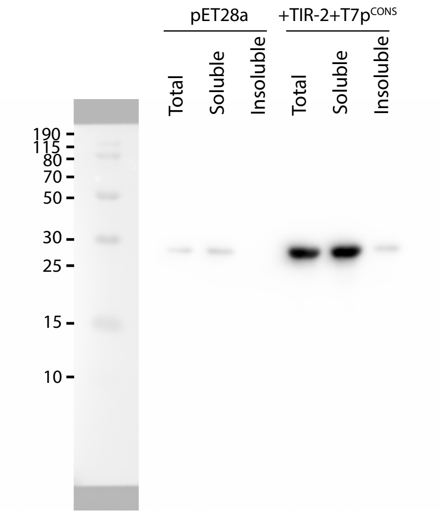

Supplementary Figure 15. Uncropped Western blots used in Figure 5a.

The presented biological replicates were used to quantify the amount of over-expressed sfGFP from fractionated BL21(DE3) *pLysS*.
All lanes are labelled according to the figure within the main text. Marker is indicated. The blue box indicates the cropped portion used in Figure 5a. Western blots detected by 1° Anti-His-HRP
a. Two replicates highlighting fractionated distribution of over-expressed sfGFP
b. One replicate highlighting fractionated distribution of over-expressed sfGFP

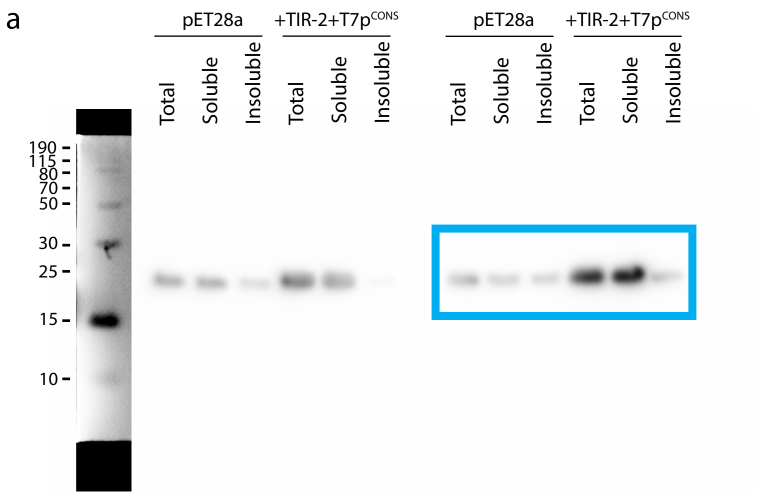

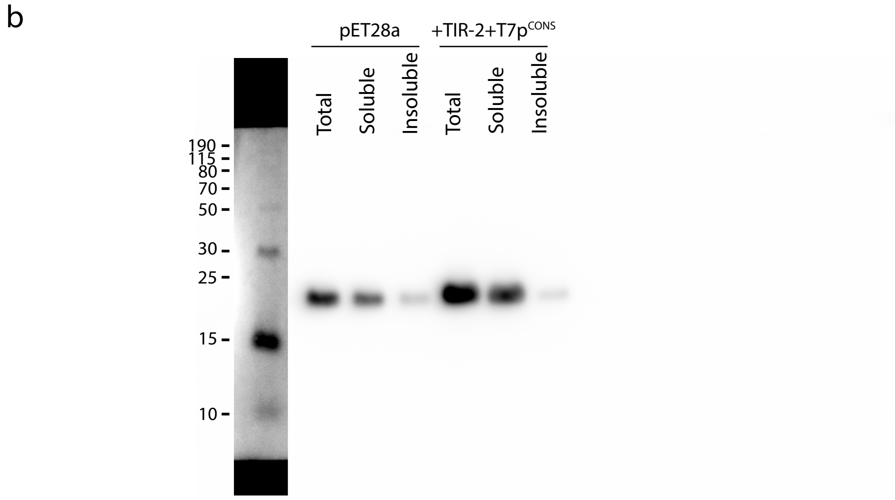

Supplementary Figure 16. Uncropped Western blots used in Figure 5b.

The presented biological replicates were used to quantify the amount of over-expressed MTH1 from fractionated BL21(DE3) *pLysS.*

All lanes are labelled according to the figure within the main text. Marker is indicated. The blue box indicates the cropped portion used in Figure 5b. Western blots detected by 1° Anti-His-HRP

a. Two replicates highlighting fractionated distribution of over-expressed MTH1

b. One replicate highlighting fractionated distribution of over-expressed MTH1

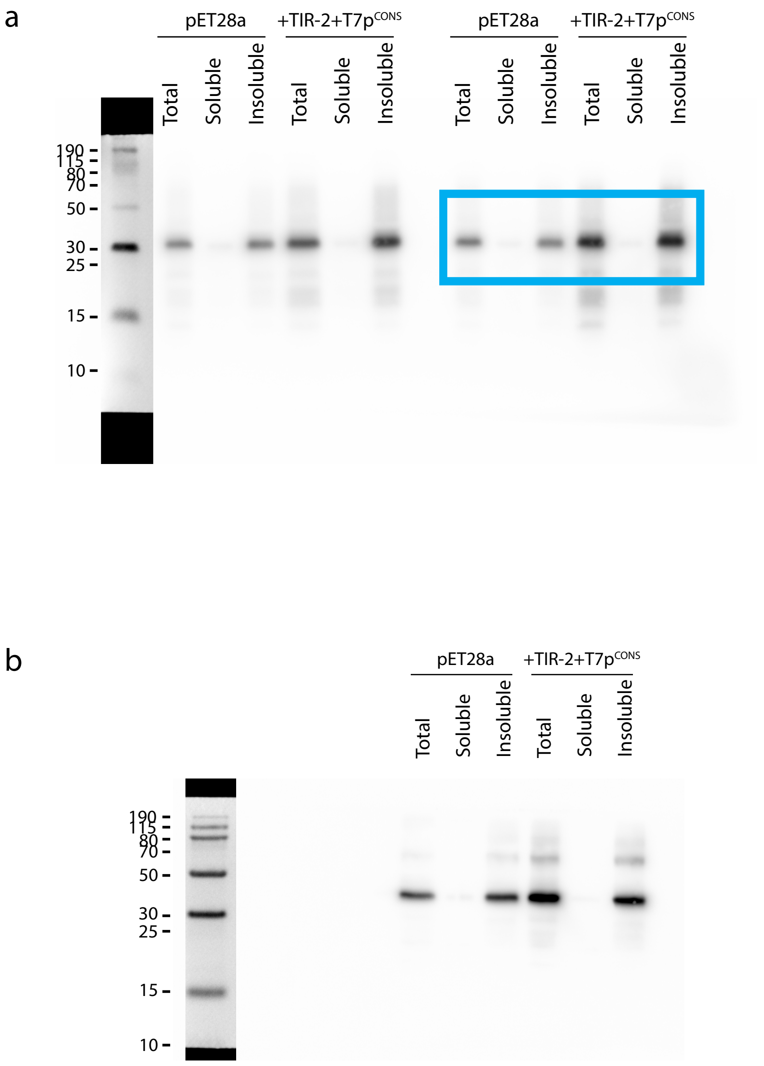

Supplementary Figure 17. Uncropped Western blots used in Figure 5c.

The presented biological replicates were used to quantify the amount of over-expressed Neil3 from fractionated BL21(DE3) *pLysS*.

All lanes are labelled according to the figure within the main text. Marker is indicated. The blue box indicates the cropped portion used in Figure 5c. Western blots detected by 1° Anti-His-HRP

a. Two replicates highlighting fractionated distribution of over-expressed Neil3

b. One replicate highlighting fractionated distribution of over-expressed Neil3

Supplementary Table 1. Oligonucleotides used for molecular cloning

Oligo name	Sequence 5'-3'
**sfGFP.Fwd**	CATAATTACATATGAGCAAAGGAGAAGAACTTTTC
**sfGFP.Rvs**	CATAATTACTCGAGTTATTTGTAGAGTTCATCCATGC
**T7pCONS.FWD**	CTATAGGGAGAGGAATTGTGAGCGGATAAC
**T7pCONS.RVS**	CCTCTCCCTATAGTGAGTCGTATTAATTTCGCGG
**Gibson oligos**	
**β-lactamase_Gibson.Fwd**	GAAATAGGAGGTCCTCCTATTTCAATTCAACATTTCCGTGTCGC
**β-lactamase_Gibson.Rvs**	CAGTGGTGGTGGTGGTGGTGCTCGAGTTACCAATGCTTAATCAGTGAG
**pET28a_sfGFP_Gibson.Rvs**	GAAATAGGAGGACCTCCTATTTCATTTGTAGAGTTCATCCATGCC
**pET28a_sfGFP_Gibson.Fwd**	CTCGAGCACCACCACCACCACCACTGAG
**pET28a.Rvs**	CATATGGCTGCCGCGCGGCACCAGGC
**Neil3_pET28a.Fwd**	AGCGGCCTGGTGCCGCGCGGCAGCCATATGGTGGAAGGACCAGGCTGTA
**Neil3_pET28a.Rvs**	ATCTCAGTGGTGGTGGTGGTGGTGCTCGAGTTATTGACAGTGAGGACAG
**Library oligos**	
**TIR_Synonymous1_FWD**	AACTTTAAGAAGGAGA**nnnnnn**ATGGG**n**AG**y**AGCCATCATCATCATCATCA
**TIR_Synonymous2_FWD**	AACTTTAAGAAGGAGA**nnnnnn**ATGGG**nTCn**AGCCATCATCATCATCATCA
**TIR_NonSynonymous_FWD**	AACTTTAAGAAGGAGA**nnnnnn**ATG**nnnnnn**AGCCATCATCATCATCATCA
**TIR_RVS**	CTCCTTCTTAAAGTTAAACAAAATTATTTCTAGAGGGGAATTGTTATC
**Back engineering oligos**	
**TIR-1.FWD**	AACTTTAAGAAGGAGAGTTATCATGGGTAGCAGCCATCATCATCATCATCA
**TIR-2.FWD**	AACTTTAAGAAGGAGAGCAGCTATGCAGCTTAGCCATCATCATCATCATCA
***In silico* oligos**	
**RBSdesigner_fwd**	AAGGAAGGTTCAAATGGGCAGCAGCCATC
**RBSdesigner_rvs1**	TTGAACCTTCCTTTTTTTGAAATAACAAAATTATTTCTAGAGGGGAATTG
**RBSdesigner_rvs2**	TTGAACCTTCCTTATCTAGAGCCAACAAAATTATTTCTAGAGGGGAATTG
**RBSdesigner_rvs3**	TTGAACCTTCCTTATCTAGAGCTAACAAAATTATTTCTAGAGGGGAATTG
**UTRdesigner_fwd1**	CAAAAGGAGGGATCAGGGAATGGGCAGCAGCCATC
**UTRdesigner_rvs1**	ATCCCTCCTTTTGGTATTCCAAAATTATTTCTAGAGGGGAATTGTT
**UTRdesigner_fwd2**	ACAAAAGTAGGGACCAGGCAATGGGCAGCAGCCATC
**UTRdesigner_rvs2**	GTCCCTACTTTTGTTAGTGCAAAATTATTTCTAGAGGGGAATTGTT
**UTRdesigner_fwd3**	AACAAAAGTAGGGACCAGGGAATGGGCAGCAGCCATC
**UTRdesigner_rvs3**	TCCCTACTTTTGTTATTGCAAAATTATTTCTAGAGGGGAATTGTT
**RBScalc_fwd1**	ATTTACTTATAAGGAGGTTTTTTATGGGCAGCAGCCATC
**RBScalc_rvs1**	CCTCCTTATAAGTAAATTGTTTAATTATTTCTAGAGGGGAATTGTTATCC
**RBScalc_fwd2**	ATTTCAAAAGGAGGTAATTTATGGGCAGCAGCCATC
**RBScalc_rvs2**	CCTCCTTTTGAAATTGATAGATTATTTCTAGAGGGGAATTGTTATCC
**RBScalc_fwd3**	ATTCACTAAGGAAACGGTTTTTTATGGGCAGCAGCCATC
**RBScalc_rvs3**	GTTTCCTTAGTGAATGTATTTAGATTATTTCTAGAGGGGAATTGTTATCC

Oligos containing blue text indicate degenerate nucleotide changes
"n" represents any nucleotide
"y" represents either a thymidine or cytosine nucleotide
