# Supplementary material for: Improved designs for pET expression plasmids increase protein production yield in Escherichia coli
Source: Commun Biol. 2020 May 7;3:214. doi: 10.1038/s42003-020-0939-8 (PMC7205610; doi:10.1038/s42003-020-0939-8)
Supplement: Supplementary file 2 — Description of Additional Supplementary Files [file 42003_2020_939_MOESM2_ESM.pdf]

Description of Additional Supplementary Files

File Name: **Supplementary Data 1**
Description: Raw data used to make figures
