# Supplementary material for: Improved designs for pET expression plasmids increase protein production yield in Escherichia coli
Source: Commun Biol. 2020 May 7;3:214. doi: 10.1038/s42003-020-0939-8 (PMC7205610; doi:10.1038/s42003-020-0939-8)
Supplement: Supplementary file 4 — Reporting Summary [file 42003_2020_939_MOESM4_ESM.pdf]

nature research

Corresponding author(s): Patrick J. Shilling, Daniel O. Daley

Last updated by author(s): Mar 6, 2020

Reporting Summary

Nature Research wishes to improve the reproducibility of the work that we publish. This form provides structure for consistency and transparency in reporting. For further information on Nature Research policies, see Authors & Referees and the Editorial Policy Checklist.

Statistics

For all statistical analyses, confirm that the following items are present in the figure legend, table legend, main text, or Methods section.

n/a	Confirmed	
☐	☒	The exact sample size (n) for each experimental group/condition, given as a discrete number and unit of measurement
☐	☒	A statement on whether measurements were taken from distinct samples or whether the same sample was measured repeatedly
☐	☒	The statistical test(s) used AND whether they are one- or two-sided<br>*Only common tests should be described solely by name; describe more complex techniques in the Methods section.*
☒	☐	A description of all covariates tested
☒	☐	A description of any assumptions or corrections, such as tests of normality and adjustment for multiple comparisons
☐	☒	A full description of the statistical parameters including central tendency (e.g. means) or other basic estimates (e.g. regression coefficient) AND variation (e.g. standard deviation) or associated estimates of uncertainty (e.g. confidence intervals)
☐	☒	For null hypothesis testing, the test statistic (e.g. F, t, r) with confidence intervals, effect sizes, degrees of freedom and P value noted<br>*Give P values as exact values whenever suitable.*
☒	☐	For Bayesian analysis, information on the choice of priors and Markov chain Monte Carlo settings
☒	☐	For hierarchical and complex designs, identification of the appropriate level for tests and full reporting of outcomes
☒	☐	Estimates of effect sizes (e.g. Cohen's d, Pearson's r), indicating how they were calculated

Our web collection on statistics for biologists contains articles on many of the points above.

Software and code

Policy information about availability of computer code

Data collection	Azure c600 imaging system<br>Odyssey imaging system<br>Spectramax Gemini<br><br>Generation of translation initiation regions were carried out with three freely available in silico prediction tools.<br>RBS calculator, version 2.1 https://salislab.net/software/predict_rbs_calculator<br>RBS designer, version 1.0.78 http://ssbio.cau.ac.kr/web/?page_id=195<br>UTR designer, online form https://sbi.postech.ac.kr/utr_designer/
Data analysis	Microsoft Excel 2016<br>ImageJ<br>Image Studio Lite<br>GraphPad Prism8 (GraphPad Software)

For manuscripts utilizing custom algorithms or software that are central to the research but not yet described in published literature, software must be made available to editors/reviewers. We strongly encourage code deposition in a community repository (e.g. GitHub). See the Nature Research guidelines for submitting code & software for further information.

Data

Policy information about availability of data

All manuscripts must include a data availability statement. This statement should provide the following information, where applicable:

- Accession codes, unique identifiers, or web links for publicly available datasets
- A list of figures that have associated raw data
- A description of any restrictions on data availability

All data supporting this study are available within the article and its Supplementary Information file or are available from the corresponding author upon request.

Field-specific reporting

Please select the one below that is the best fit for your research. If you are not sure, read the appropriate sections before making your selection.

☒ Life sciences ☐ Behavioural & social sciences ☐ Ecological, evolutionary & environmental sciences

For a reference copy of the document with all sections, see nature.com/documents/nr-reporting-summary-flat.pdf

Life sciences study design

All studies must disclose on these points even when the disclosure is negative.

Sample size	No statistical methods were used to predetermine sample size. Three biological replicates were used in each experiment, allowing data reproducibility.
Data exclusions	No data exclusions apply to this study
Replication	All replicates contained within this study are represented as biological replicates. Biological replicates are defined in this study as individual colonies of E. coli strains (either BL21(DE3)pLysS, C41 or C43) transformed with defined plasmids.
Randomization	Not applicable
Blinding	Not applicable

Reporting for specific materials, systems and methods

We require information from authors about some types of materials, experimental systems and methods used in many studies. Here, indicate whether each material, system or method listed is relevant to your study. If you are not sure if a list item applies to your research, read the appropriate section before selecting a response.

Materials & experimental systems

n/a	Involved in the study	
☐	☒	Antibodies
☒	☐	Eukaryotic cell lines
☒	☐	Palaeontology
☒	☐	Animals and other organisms
☒	☐	Human research participants
☒	☐	Clinical data

Methods

n/a	Involved in the study	
☒	☐	ChIP-seq
☒	☐	Flow cytometry
☒	☐	MRI-based neuroimaging

Antibodies

Antibodies used	1. HisProbe™-HRP Conjugate<br>- Catalog# 15165<br>- Lot# RE233744<br>2. Anti-MTH1 Antibody<br>- Catalog# MABC1040<br>- Clone 11A3.2<br>- Lot# Q2672610
Validation	1. HisProbe™-HRP Conjugate (Details from Certificate of Analysis)<br>Cross reactivity: All species<br>Immunogen: His-tagged proteins or proteins possessing stretches of histidine residues<br>Specificity: His-tagged proteins and other histidine-rich proteins in Western blots<br>Validation: https://assets.thermofisher.com/TFS-Assets/LSG/figures/hisprobe-hrp-reagent-kit-fig.jpg-650.jpg

2. Anti-MTH1 (Details from Certificate of Analysis)
Species Cross-reactivity: Human
Immunogen: GST-tagged recombinant human MTH1 C-terminal fragment.
Specificity: Clone 11A3.2 recognizes an epitope present in all four spliced isoforms of human NUDT1/MTH1 reported by UniProt (P36639).
Quality Assurance/Validation: Evaluated by Western Blotting in Jurkat cell lysate. Western Blotting Analysis: 1.0 µg/mL of this antibody detected MTH1 in 10 µg of Jurkat cell lysate.
